# Supplementary material for: Adaptation and Validation of the Turkish Version of the Brain Fog Scale
Source: Int J Environ Res Public Health. 2024 Jun 14;21(6):774. doi: 10.3390/ijerph21060774 (PMC11203739; doi:10.3390/ijerph21060774)
Supplement: Supplementary file 1 [file ijerph-21-00774-s001.zip › ijerph-3004067-supplementary.pdf]

Supplementary Table 1. Descriptive statistics of the demographic, health, and nutritional findings of individuals included in pilot study

	**Man** (*n*=27)		**Woman** (*n*=98)		**Total** (*n*=125)	
	**n**	**%**	**n**	**%**	**n**	**%**
**Age (year) ($\bar{X} \pm SD$)**	34,26±7,11		35,87±7,71		35,52±7,59	
**Educational Level**						
Primary school	0	0,0	1	1,0	1	0,8
Secondary school	0	0,0	2	2,0	2	1,6
High school	6	22,2	10	10,2	16	12,8
Bachelor degree	16	59,3	59	60,2	75	60,0
MSc and PhD	5	18,5	26	26,5	31	24,8
**Occupation**						
Civil servant	7	25,9	23	23,5	30	24,0
Private sector	6	22,3	40	40,8	46	36,8
Self-employment	9	33,3	11	11,2	20	16,0
Retired	0	0,0	1	1,0	1	0,8
Housewife	0	0,0	3	3,1	3	2,4
Student	4	14,8	1	1,0	5	4,0
Unemployed	1	3,7	19	19,4	20	16,0
**Chronic Disease Diagnosed by a Doctor**						
Yes	5	18,5	37	37,8	42	33,6
No	22	81,5	61	62,2	83	66,4
**Skipping Meals**						
I don't skip meals	3	11,1	14	14,3	17	13,6
Sometimes I skip meals	12	44,4	49	50,0	61	48,8
I skip breakfast	9	33,4	15	15,3	24	19,2
I skip lunch	3	11,1	20	20,4	23	18,4
**BMI Classification**						
Underweight	1	3,7	1	1,0	2	1,6
Normal	9	33,3	60	61,3	69	55,2
Overweight	15	55,6	25	25,5	40	32,0
Obese	2	7,4	12	12,2	14	11,2

**BMI (kg/m²) ($\bar{X} \pm SD$)**	25,82±3,80		24,55±4,19		24,82±4,13	
**Thinking of Eating Healthy**						
Yes	15	55,6	53	54,1	68	54,4
No	12	44,4	45	45,9	57	45,6
**Physical Activity Status in the Last Week**						
I did it for 1 day	16	59,3	50	51,0	66	52,8
I did it for 2 day	5	18,5	17	17,3	22	17,6
I did it for 3 day	1	3,7	16	16,3	17	13,6
I did it for 4 day	2	7,4	8	8,2	10	8,0
I did it for 5 day	3	11,1	3	3,1	6	4,8
I did it for 6 day	0	0,0	3	3,1	3	2,4
I did it for 7 day	0	0,0	1	1,0	1	0,8
**Body Weight Assessment Status**						
Underweight	1	3,7	4	4,1	5	4,0
Normal	16	59,3	51	52,0	67	53,6
Overweight	6	22,2	33	33,7	39	31,2
Obese	4	14,8	10	10,2	14	11,2
**General Health Evaluation Status**						
Poor	0	0,0	2	2,0	2	1,6
Fair	6	22,3	23	23,5	29	23,2
Good	13	48,1	58	59,2	71	56,8
Very good	8	29,6	14	14,3	22	17,6
Excellent	0	0,0	1	1,0	1	0,8
**COVID-19 Diagnosis Status**						
Yes	12	44,4	59	60,2	71	56,8
No	15	55,6	39	39,8	54	43,2
